# Characterization of a highly diverged mitochondrial ATP synthase F_o_ subunit in *Trypanosoma brucei*

**DOI:** 10.1016/j.jbc.2022.101829

**Published:** 2022-03-12

**Authors:** Caroline E. Dewar, Silke Oeljeklaus, Christoph Wenger, Bettina Warscheid, André Schneider

**Affiliations:** 1 Department of Chemistry, Biochemistry and Pharmaceutical Sciences, University of Bern, Bern, Switzerland; 2 Department of Biochemistry, Theodor Boveri-Institute, University of Würzburg, Würzburg, Germany; 3 CIBSS Centre for Integrative Biological Signalling Studies, University of Freiburg, Freiburg, Germany

**Keywords:** ATP synthase, mitochondria, *Trypanosoma brucei*, proteomics, protozoan, BN-PAGE, blue native PAGE, BSA, bovine serum albumin, CM-H_2_DCFHDA, chloromethyl derivative of H_2_DCFHDA, IF, immunofluorescence, IM, inner membrane, IMS, intermembrane space, IP, immunoprecipitation, MS, mass spectrometry, OSCP, oligomycin sensitivity–conferring protein, ROS, reactive oxygen species, SILAC, stable isotope labeling by amino acids in cell culture, TMD, transmembrane domain, TMRE, tetramethylrhodamine ethyl ester, VDAC, voltage-dependent anion channel

## Abstract

The mitochondrial F_1_F_o_ ATP synthase of the parasite *Trypanosoma brucei* has been previously studied in detail. This unusual enzyme switches direction in functionality during the life cycle of the parasite, acting as an ATP synthase in the insect stages, and as an ATPase to generate mitochondrial membrane potential in the mammalian bloodstream stages. Whereas the trypanosome F_1_ moiety is relatively highly conserved in structure and composition, the F_o_ subcomplex and the peripheral stalk have been shown to be more variable. Interestingly, a core subunit of the latter, the normally conserved subunit *b*, has been resistant to identification by sequence alignment or biochemical methods. Here, we identified a 17 kDa mitochondrial protein of the inner membrane, Tb927.8.3070, that is essential for normal growth, efficient oxidative phosphorylation, and membrane potential maintenance. Pull-down experiments and native PAGE analysis indicated that the protein is both associated with the F_1_F_o_ ATP synthase and integral to its assembly. In addition, its knockdown reduced the levels of F_o_ subunits, but not those of F_1_, and disturbed the cell cycle. Finally, analysis of structural homology using the HHpred algorithm showed that this protein has structural similarities to F_o_ subunit *b* of other species, indicating that this subunit may be a highly diverged form of the elusive subunit *b*.

Most cellular ATP is synthesized by the F_1_F_o_ ATP synthase complex which, in eukaryotic cells, is localized to mitochondria and plastids. The canonical mitochondrial F_1_F_o_ ATP synthase consists of two subcomplexes, the membrane-embedded F_o_ and the soluble F_1_ portions, which are connected by both a central stalk that rotates within the F_1_ moiety and a stationary peripheral stalk (Fig. 1). The F_1_ moiety contains a hexamer of alternating α subunits and β subunits surrounding a single γ subunit, which functions as the central stalk. The γ subunit protrudes out from this headpiece to connect with the F_o_ moiety. The F_o_ moiety holds the proton translocation channel through the inner mitochondrial membrane formed by subunit *a* and a *c* subunit oligomer in a ring conformation. The F_1_ subcomplex is generally conserved in terms of catalytic mechanism, inhibition of the reverse reaction by IF_1_, subunit stoichiometry, subunit sequences, and structure. Proton movement through the F_o_ moiety rotates the *c*-ring and the central γ subunit, which induces asymmetrical conformational changes in the three active sites on the α subunit and β subunit interfaces, resulting in the stepwise ATP synthesis cycle (1).

**Figure 1:**
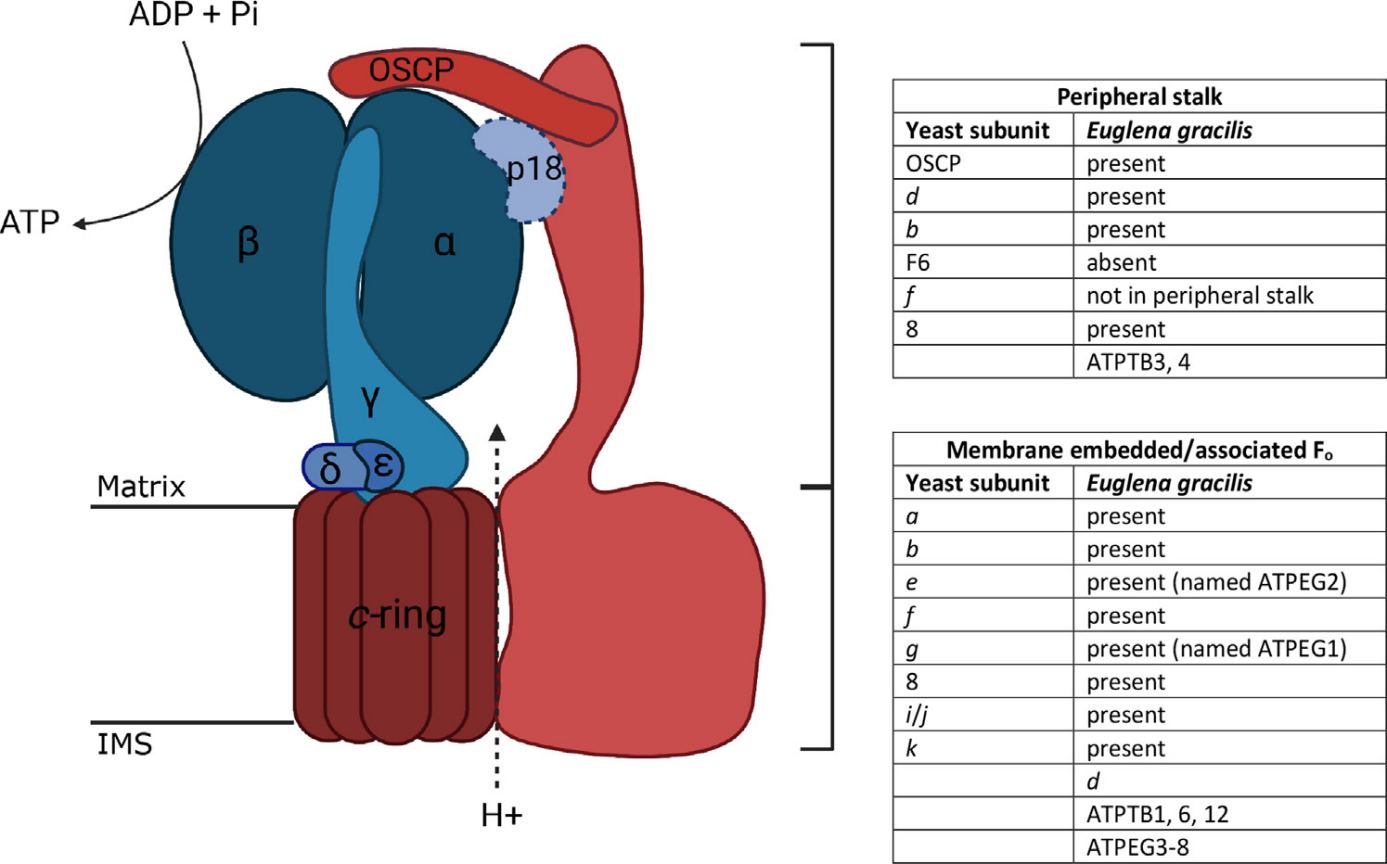
**Schematic depicting the structure and function of a typical mitochondrial F**_**1**_**F**_**o**_**ATP synthase complex.** A schematic showing ATP synthase subunit composition in yeast, with a comparison to the subunits found in the *Euglena gracilis* structure and their location. ATP synthesis in the F_1_ catalytic head (*blue*) is coupled to proton translocation through the membrane-embedded F_o_ portion (*red*). The protons are translocated between subunit *a* and the *c*-ring, driving the rotation of the *c*-ring and the central rotor, made up of subunits γ, ε, and δ. Asymmetric interactions of subunit γ during this rotation force conformational changes in catalytic subunits α and β and thereby induce ATP synthesis. Futile rotation of the α/β headpiece is prevented by OSCP and the peripheral stalk, which holds the external parts of the F_1_ moiety stationary. The subunits labeled on the scheme, along with proton-translocating subunit *a*, are found in all eukaryotic lineages, with the exception of p18, which is exclusively found in Euglenozoa. Schematic created with BioRender.com. IMS, intermembrane space; OSCP, oligomycin sensitivity–conferring protein.

The catalytic hexamer is prevented from rotation with the central stalk and the *c*-ring by the peripheral stalk, which acts as a stator. This is essential for the driving of conformational changes in the α_3_β_3_ hexamer and for the coupling of proton movement with ATP synthesis. In the yeast and mammalian enzymes, the conserved oligomycin sensitivity–conferring protein (OSCP), part of the peripheral stalk, sits at the pinnacle of the F_1_ head and interacts with the α subunit, *h*/F_6_ subunit, and *b* subunits (2, 3, 4, 5, 6). The central component of the peripheral stalk is subunit *b*, which stretches from the OSCP to the membrane-bound F_o_ moiety, and interacts with subunits *d* and *h*/F_6_ *via* stable coiled coils (5). There is also direct contact between a catalytic α subunit and subunits *b*, *d*, and *h*/F_6_ (7). In the membrane, subunit *b* interacts with subunits 8 and *d* (8, 9), the proton pore subunit *a* (6, 7, 9, 10, 11), subunit *f* (10), and the dimerization subunits *e* and *g* (9).

Despite overall structural and functional conservation between F_1_F_o_ ATP synthases of all three domains (12), there is now evidence for substantial divergence between eukaryotic lineages in proteins making up the noncore parts of the complex (Fig. 1) (13, 14, 15, 16, 17, 18, 19, 20). Subunits of the peripheral stalk and subunits involved in the anchorage of the peripheral stalk to the membrane, in holding the F_o_ *a* subunit in proximity to the *c*-ring and in the dimerization domains, all show in general low-sequence conservation between species. However, while many noncore ATP synthase subunits show no sequence similarity between species, structural homology can sometimes still be detected (13, 14, 21).

Recently, the unusual functioning of the mitochondrial F_1_F_o_ ATP synthase in the complex life cycle of the parasitic protozoan *Trypanosoma brucei* has attracted a lot of interest (14, 22). During the tsetse midgut stages of the life cycle, the complex generates ATP *via* oxidative phosphorylation using substrates derived from amino acid catabolism. Similar to other eukaryotes, trypanosomal F_1_F_o_ ATP synthase dimers are thought to facilitate cristae formation and may increase the efficiency of oxidative phosphorylation (23, 24). In the mammalian bloodstream forms of the parasite however, although still functioning in Fe/S cluster assembly, metabolism, and ion homeostasis, the mitochondrion is reduced in volume and does not express a cytochrome-dependent electron transport chain or carry out oxidative phosphorylation (22). ATP is generated primarily through glycolysis, and unusually, the F_1_F_o_ ATP synthase acts in reverse as an ATPase, hydrolyzing mitochondrial ATP to generate the essential membrane potential (ΔΨm) (25, 26, 27, 28, 29). Despite the importance of this role, the *T. brucei* mitochondrion is remarkably tolerant to both the level of F_1_F_o_ ATPase complexes present (30) and to alterations in the F_1_F_o_ ATPase structure (21, 25, 31).

The composition and structure of the F_1_F_o_ ATP synthase of procyclic *T. brucei* and of *Euglena gracilis* have been analyzed in detail (13, 14, 23, 32, 33, 34, 35). Trypanosomes and *Euglena* belong to the euglenozoans and thus are phylogenetically quite closely related. The architecture of the catalytic F_1_ moiety of both complexes from the two species is essentially canonical (34), containing the conserved subunits α, β, γ, δ, and ε (33). Unusually, the α subunits of both are found cleaved into two chains (31, 36), an euglenozoan-specific feature of unknown biological relevance (37, 38, 39). The F_1_ headpiece also contains three copies of an additional euglenozoan-specific subunit, termed p18. This protein was first thought to be subunit *b* of the peripheral stalk (33, 39, 40) but has now been shown to be an F_1_ subunit that is associated with the α subunits. It facilitates the interaction between the F_1_ moiety and the peripheral stalk (34, 36).

In the F_o_ portion of these complexes, subunits ATPTB1, ATPTB3, ATPTB4, ATPTB6, and ATPTB12 are deemed to be euglenozoan specific (13). There is to date no structural information on the F_o_ moiety including the peripheral stalk of the *T. brucei* enzyme complex. However, the structure of the peripheral stalk of F_1_F_o_ ATP synthase of *E. gracilis* has been defined and is highly unusual. It features an extended OSCP and a divergent *d* subunit homolog termed ATPTB2 that contacts the ATPTB3, ATPTB4 and p18 subunits to prevent futile F_1_ head rotation, substituting for a reduced subunit *b* (13).

BLAST analysis using the sequences of the peripheral stalk subunit *b* from either yeast, mammals or from *E. gracilis* as templates failed to identify a trypanosomal homolog, and it has therefore been speculated that the trypanosomal ortholog might be either highly reduced in size as in *E. gracilis* and therefore difficult to find or that it might even be absent (14). Here, we show by using a combination of biochemical, molecular, genetic, and *in silico* analyses that the *T. brucei* F_1_F_o_ ATP synthase likely contains an unusual F_o_ subunit that shows similarities to subunit *b* of other species.

## Results

### Tb927.8.3070 is an integral mitochondrial inner membrane protein

Recently, we analyzed the mitochondrial proteome of the parasitic protozoan *T. brucei* using the ImportOmics approach. The resulting proteome consisted of 1120 proteins, many of which are of unknown function and do not have orthologs outside the kinetoplastids (41). In the present study, we are focusing on one of these proteins, Tb927.8.3070, which is 145 amino acids in length and has a molecular mass of 17 kDa. Since the protein contains a single predicted transmembrane domain (TMD), but is not part of the previously characterized mitochondrial outer membrane proteome (42), it was assumed to be an integral inner membrane (IM) protein.

**Figure 2:**
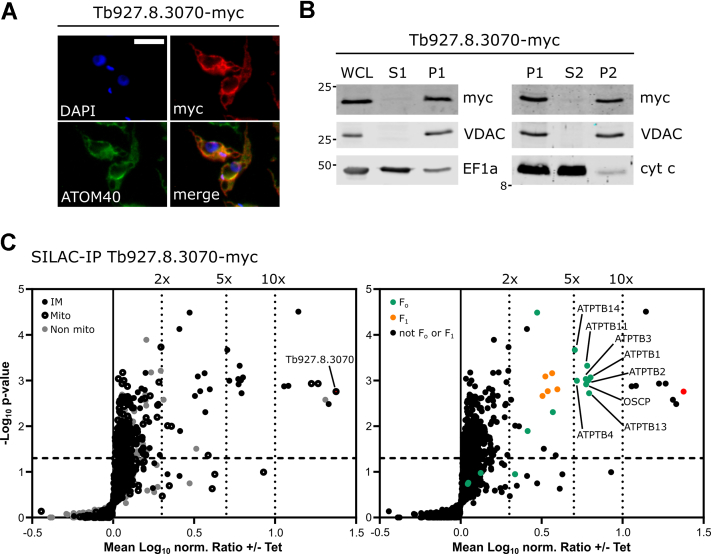
**Tb927.8.3070 is an integral mitochondrial inner membrane protein that interacts with the F**_**1**_**F**_**o**_**ATP synthase complex.***A*, IF image of a procyclic *Trypanosoma brucei* cell line allowing tetracycline-inducible expression of Tb927.8.3070-myc. ATOM40 serves as a mitochondrial marker. DAPI marks both nuclear and mitochondrial DNA. The scale bar represents 10 μm. Cells were induced overnight. *B*, subcellular fractionation of cells expressing Tb927.8.3070-myc. *Left panel*, immunoblot analysis of whole cell lysates (WCLs) and digitonin-extracted mitochondria-enriched (P1) and soluble cytosolic (S1) fractions of cells expressing Tb927.8.3070-myc. The immunoblots were probed with anti-myc antibodies and antisera against VDAC and elongation factor 1-alpha (EF1a), which serve as markers for mitochondria and cytosol, respectively. *Right panel*, digitonin-extracted mitochondria-enriched fractions (P1) were subjected to alkaline carbonate extraction performed at pH 11.5 resulting in membrane-enriched pellet (P2) and soluble supernatant (S2) fractions. Subsequent immunoblots were probed with anti-myc and antisera against VDAC and cytochrome *c* (cyt *c*), which serve as markers for integral and peripheral membrane proteins, respectively. *C*, volcano plots of a SILAC–IP analysis of crude mitochondrial extracts from Tb927.8.3070-myc–expressing cells. Cells were induced with tetracycline for 1 day. Proteins were quantified in three independent biological replicates, with the mean log_2_ of ratios (with/without Tet) plotted against the −log_2_*p* value (two-sided *t* test). The bait is shown in *red*. The *horizontal dashed line* shows a significance level of *p* = 0.05. The *vertical dotted lines* mark specified enrichment factors. *Left panel*, the following groups of proteins are highlighted: mitochondrial IM proteins (IM, *black*), other proteins of the mitochondrial importome (Mito, *black with a white center*), and non mitochondrial proteins (non mito, *gray*). *Right panel*, the following groups of proteins are highlighted: ATP synthase F_o_ subunits (F_o_, *orange*) and F_1_ subunits (F_1,_*green*). ATOM40, atypical translocase of the outer membrane 40; DAPI, 4′,6-diamidino-2-phenylindole; IF, immunofluorescence; IM, inner membrane; SILAC–IP, stable isotope labeling by amino acids in cell culture–immunoprecipitation; VDAC, voltage-dependent anion channel.

In order to test this prediction, we produced a transgenic *T. brucei* cell line allowing tetracycline-inducible expression of C-terminally myc-tagged Tb927.8.3070. Immunofluorescence (IF) analysis of tetracycline-induced cells using anti-myc antibodies indicated that myc-tagged Tb927.8.3070 colocalized with the mitochondrial marker atypical translocase of the outer membrane 40 (Fig. 2*A*). In addition, cell extractions with 0.015% digitonin showed that myc-tagged Tb927.8.3070 cofractionates with the voltage-dependent anion channel (VDAC), which serves as another mitochondrial marker (Fig. 2*B*, *left panel*). The tagged protein was also exclusively recovered in the pellet when the crude mitochondrial fraction was subjected to carbonate extraction at high pH (Fig. 2*B*, *right panel*), along with the integral outer membrane protein VDAC. This suggests that Tb927.8.3070 is an integral mitochondrial membrane protein consistent with its expected localization in the mitochondrial IM, even though no N-terminal mitochondrial targeting sequence can be reliably predicted using multiple algorithms.

### Tb927.8.3070 interacts with the F_1_F_o_ ATP synthase complex

To analyze whether Tb927.8.3070 was contained within a protein complex, a transgenic *T. brucei* cell line allowing inducible expression of Tb927.8.3070-myc was subjected to stable isotope labeling by amino acids in cell culture (SILAC) followed by an anti-myc immunoprecipitation (IP) from mitochondria-enriched fractions. The resulting eluates were analyzed by quantitative mass spectrometry (MS). Including the myc-tagged Tb927.8.3070, which served as bait, 30 proteins were found significantly more than twofold enriched in this IP (Fig. 2*C* and Table S1). Twenty-five of these proteins were members of the mitochondrial importome (41) (Fig. 2*C*, *left panel*), with 19 designated as being mitochondrial IM proteins (42). Strikingly, 16 previously identified subunits of the F_1_F_o_ ATP synthase (33) were significantly enriched more than twofold (Fig. 2*C*, *right panel*), suggesting that tagged Tb927.8.3070 might be a component of or interact with the F_1_F_o_ ATP synthase. Interestingly, the F_1_F_o_ ATP synthase subunits that were found most highly enriched (between 5-fold–10-fold) included OSCP and ATPTB2, known subunits of the peripheral stalk, and ATBTB1, ATBTB3, ATBTB4, ATBTB11, ATBTB13, and ATBTB14 of the F_o_ moiety.

Other proteins that were found significantly more than 10-fold enriched include a M76 peptidase with homology to yeast and mammalian ATP23, which is a protease of the IM required for F_1_F_o_ ATP synthase assembly (43, 44), phosphatidylserine decarboxylase (45), and cardiolipin-dependent protein 17 (46). The remaining four proteins are kinetoplastid-specific proteins of unknown function (Fig. S1).

### Tb927.8.3070 is an essential protein and comigrates with the F_1_F_o_ ATP synthase monomer

**Figure 3:**
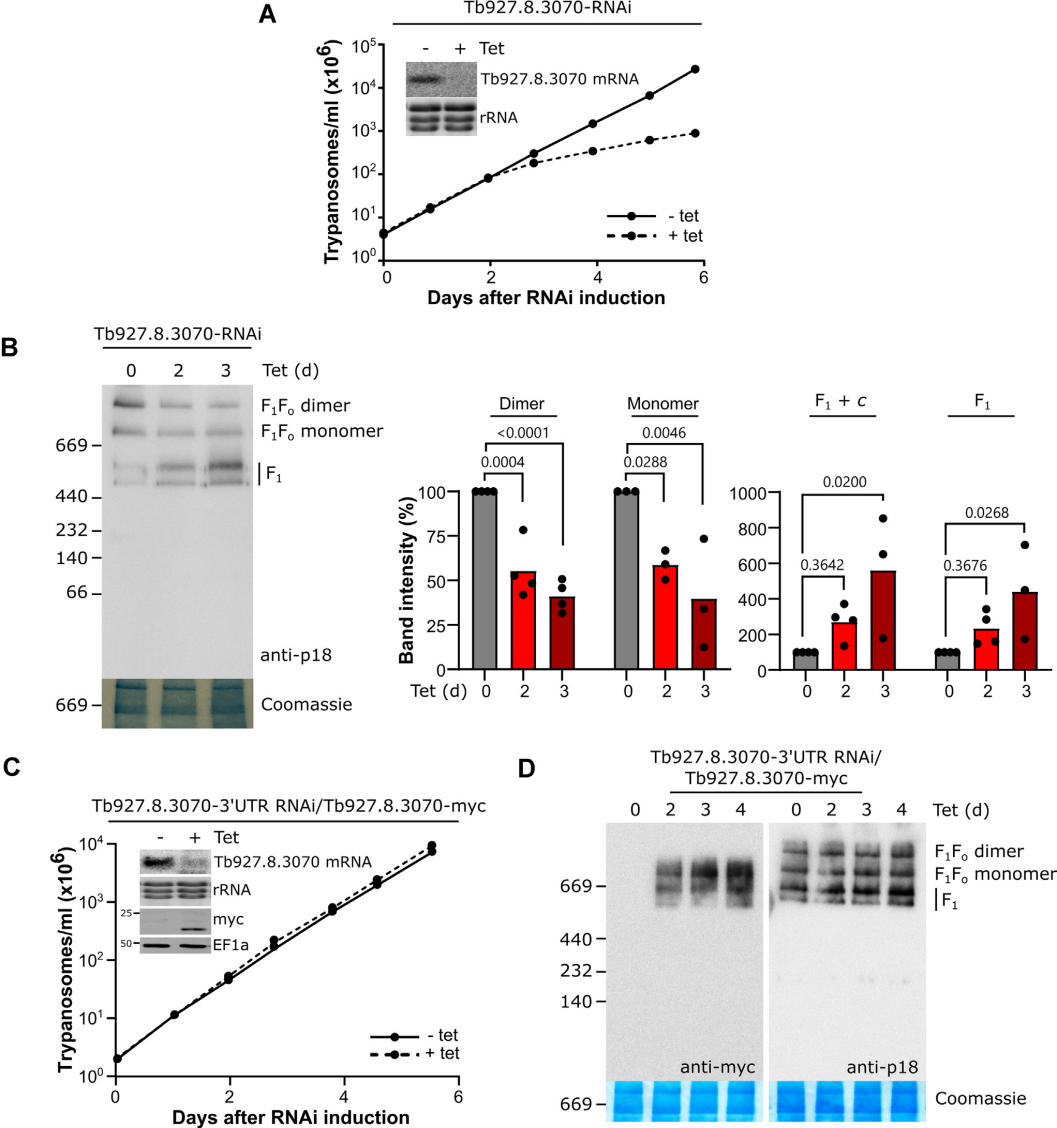
**Tb927.8.3070 is an essential protein and comigrates with the F**_**1**_**F**_**o**_**ATP synthase monomer.***A*, growth curve analysis of a procyclic Tb927.8.3070-RNAi cell line. Analysis was performed in triplicate, with error bars showing standard deviation (too small to be visible). *Inset*, Northern analysis after 2 days of induction of the level of Tb927.8.3070 mRNA. EtBr-stained rRNAs act as a loading control. *B*, *left panel*, BN-PAGE analysis of crude mitochondrial extracts of Tb927.8.3070-RNAi cells. ATP synthase complexes were visualized with a polyclonal antibody against ATP synthase F_1_ subunit p18. A section of the Coomassie-stained gel serves as a loading control. *Right panel*, quantification of the BN-PAGE using ImageJ. Three or four biological replicates were analyzed. *p* Values were calculated using a one-way ANOVA with Dunnett’s multiple comparison post hoc test. *C*, growth curve analysis of the Tb927.8.3070-RNAi 3′ UTR cell line expressing Tb927.8.3070-myc. The RNAi construct in this cell line targets the 3′ UTR of Tb927.8.3070 and therefore allows reexpression of the protein in a different genomic context. Analysis was performed in triplicate, with error bars showing standard deviation. *Inset*, Northern analysis after 2 days of induction of the level of Tb927.8.3070 mRNA and the corresponding immunoblot analysis of the Tb927.8.3070-myc levels. EtBr-stained rRNA species and EF1a act as a loading control. *D*, BN-PAGE analysis of crude mitochondrial extracts from uninduced and induced cells used in *C*. Tb927.8.3070-myc and ATP synthase complexes were visualized either with anti-myc or anti-p18. A section of the Coomassie-stained gel serves as a loading control. BN-PAGE, blue native PAGE; EF1a, elongation factor 1-alpha.

To investigate the function of Tb927.8.3070, we produced a tetracycline-inducible RNAi cell line of procyclic *T. brucei*. Knockdown of Tb927.8.3070 caused a growth retardation by day 2 postinduction (Fig. 3*A*). Next, we analyzed the consequences of Tb927.8.3070 depletion on the F_1_F_o_ ATP synthase complex and subcomplexes. Blue native PAGE (BN-PAGE) and subsequent immunoblot analysis using an antibody against F_1_ ATP synthase subunit p18 (33, 47, 48) in Tb927.8.3070-RNAi cells showed that the levels of F_1_F_o_ dimer and monomer were steadily decreased upon Tb927.8.3070 depletion to less than around 40% of the levels seen in uninduced cells by day 3 postinduction (Fig. 3*B*). Interestingly, a similar, albeit stronger, phenotype was previously seen upon depletion of F_o_ subunit ATPTB1 and peripheral stalk subunits OSCP and ATPTB2 (21, 30, 33). In contrast, the levels of the free F_1_ moiety doublet were increased up to fourfold by day 3 after Tb927.8.3070-RNAi induction (Fig. 3*B*). This doublet likely represents the core F_1_ subcomplex with and without *c*-ring attached, as in mammalian cells (21, 33, 49), although this has not been experimentally verified. These results are similar to those previously obtained after OSCP, ATPTB1, and ATPTB2 depletion (30, 33), and contrast with the effect of F_1_ subunit depletion, which reduces the level of both F_1_F_o_ and F_1_ ATP synthase complexes (33, 36). To exclude off-target effects in the Tb927.8.3070-RNAi cell line and to demonstrate that the tagged Tb927.8.3070 is functional, a cell line was generated where Tb927.8.3070 was downregulated by targeting its 3′UTR for RNAi upon tetracycline induction while a C-terminally myc-tagged version of Tb927.8.3070 was ectopically expressed. Expression of Tb927.8.3070-myc fully restored growth of the induced RNAi cell line (Fig. 3*C*). BN-PAGE analysis of crude mitochondrial extracts from this induced cell line revealed the characteristic four-band pattern of the *T. brucei* F_1_F_o_ ATP synthase that could be detected by an antibody specific for p18 was maintained upon the expression of Tb927.8.3070-myc (Fig. 3*D*, *right lane*). From this, we can conclude that myc-tagged Tb927.8.3070 can complement the growth defect caused by endogenous Tb927.8.3070 ablation. When the same samples were probed with anti-myc antibodies, multiple bands were detected (Fig. 3*D*, *left lanes*), the predominant one at the approximate size of the F_1_F_o_ ATP synthase monomer, suggesting that Tb927.8.3070-myc is present in this complex. Intriguingly, no myc signal was detected in the band corresponding to the F_1_F_o_ ATP synthase dimer. This is surprising since the level of the dimer, as detected by p18 antibodies, was restored in the complemented cell line (Fig. 3, *B* and *D*). A possible explanation for this result is that steric masking of the myc epitope in the dimer might have prevented its detection. Alternatively, Tb927.8.3070 might be a late assembly factor that is present in a complex that in size essentially coincides with the F_1_F_o_ ATP synthase monomer but whose absence precludes the formation of the dimer.

In summary, these results show that depletion of Tb927.8.3070 has a similar effect to the depletion of ATPTB1, OSCP, and ATPTB2 (21, 30, 33), and therefore is presumably not part of the F_1_ moiety but rather a subunit of the F_o_ ATP synthase subcomplex or the peripheral stalk associated with it.

### Tb927.8.3070 shows similarity to F_1_F_o_ ATP synthase subunit *b*

Interestingly, the core component of the peripheral stalk, subunit *b*, has not been identified in *T. brucei*. This is somewhat surprising since the composition of trypanosomal F_1_F_o_ ATP synthase has been analyzed in detail using proteomics (33) and a highly diverged subunit *b* had been found in the phylogenetically related *E. gracilis* (13). However, reciprocal BLAST searches using either the *E. gracilis* subunit *b* or Tb927.8.3070 as templates did not retrieve either protein or any F_1_F_o_ ATP synthase subunit of other species. It should be taken into consideration though that the two proteins are very small, 17 kDa for Tb927.8.3070 and 12.7 kDa for *Euglena* subunit *b*, which would make it difficult to detect homology of diverged sequences. In fact, without structural information on the peripheral stalk, it would not have been possible to identify the highly diverged subunit *b* of *E. gracilis*.

**Figure 4:**
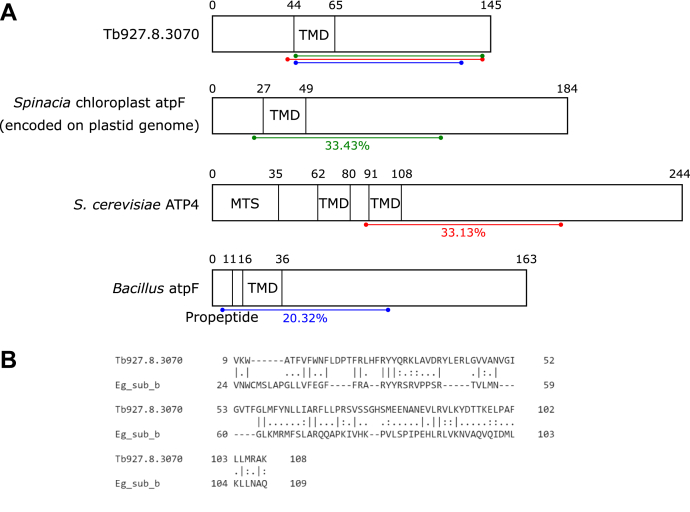
**Tb927.8.3070 shows similarities to F**_**1**_**F**_**o**_**ATP synthase subunit *b.****A*, a schematic showing a comparison of domains predicted in Tb927.8.3070 and the homologs of ATP synthase subunit *b* in spinach chloroplasts (atpF), yeast (ATP4), and *Bacillus* species (atpF). The regions of secondary structure homology as determined by HHpred are indicated by the colored lines. TMDs were predicted by HMMTOP and TMPred (86, 87). The yeast ATP4 mitochondrial targeting sequence (MTS) was previously defined experimentally (88). *B*, an alignment of the protein sequences of Tb927.8.3070 and *Euglena gracilis* subunit *b* using the EMBOSS Water Pairwise Sequence Alignment tool (89). TMD, transmembrane domain.

Thus, we wanted to investigate the possibility that Tb927.8.3070 might be the F_o_ subunit *b*. We used the HHpred algorithm that is based on the comparison of profile hidden Markov models, which often is able to establish connections to remotely homologous characterized proteins (50). The HHpred analysis retrieved 45 proteins of known function, which shared low sequence and structural similarity with Tb927.8.3070 (Fig. S2*A*). Eleven of these had regions of significant structural similarity of more than 80 amino acids. Most interestingly, four of them were related to mitochondrial ATP synthase subunit *b*: the spinach chloroplast subunit atpF, the yeast subunit ATP4, and the atpF subunits of two bacterial species, *Mycobacteria* and *Bacillus* (Fig. 4*A* and S2*A* highlighted in *blue*, Fig. S2*B*). Strikingly, in all four cases, the region showing similarity with Tb927.8.3070 has the same relative position, including the experimentally confirmed TMDs and an approximately 60 amino acid C-terminal flanking sequence of the four F_1_F_o_ ATP subunit *b* proteins (Fig. 4*A*). Tb927.8.3070 is a shorter protein, with the spinach, yeast, and bacterial proteins having a longer C-terminal extension past the region of structural similarity. BLAST detects Tb927.8.3070 sequence homologs only in kinetoplastids (Fig. S2*C*).

It is somewhat surprising that HHpred analysis of Tb927.8.3070 did not retrieve subunit *b* in the relatively closely related *Euglena*. However, a pairwise alignment of the *E. gracilis* subunit *b* protein sequence with the trypanosomal protein showed that a region covering the central 70% of Tb927.8.3070 has 23.6% identity to the *E. gracilis* protein, suggesting that the proteins do share some limited sequence conservation (Fig. 4*B*). While this similarity is low, there are homologous F_o_F_1_ ATP synthase subunits that share less sequence identity between *Euglena* and *T. brucei* (14).

### Tb927.8.3070 knockdown selectively depletes F_o_ ATP synthase subunits

**Figure 5:**
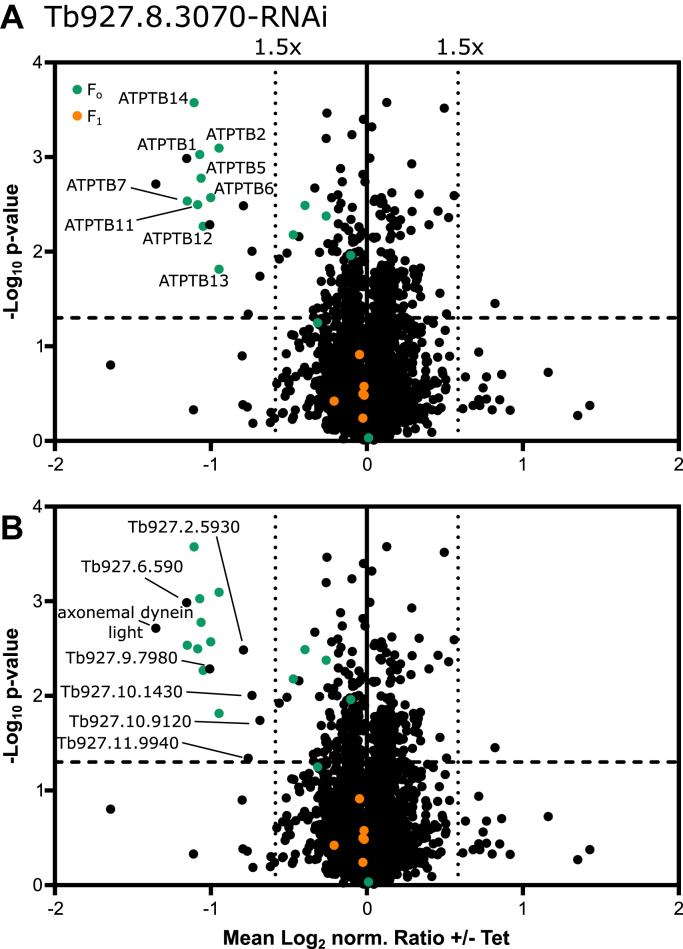
**Tb927.8.3070 depletion selectively depletes F**_**o**_**ATP synthase subunits.***A*, volcano plots of a SILAC-based quantitative MS analysis of crude mitochondrial extracts of uninduced and induced Tb927.8.3070-RNAi cells. Cells grown in SILAC media were harvested after 4 days of induction. Proteins were quantified in three independent biological replicates, with the mean log_2_ of normalized (norm.) ratios plotted against the −log_2_*p* value (two-sided *t* test). Tb927.8.3070, the RNAi target, was not detected in this analysis. The *horizontal dashed line* indicates a significance level of *p* = 0.05. The *vertical dotted lines* mark proteins with a 1.5-fold change in abundance compared with control cells. The following groups of proteins are highlighted: ATP synthase F_o_ subunits (F_o_, *orange*) and F_1_ subunits (F_1,_*green*). *B*, as in *A*, but proteins downregulated more than 1.5-fold are labeled with either their name or accession numbers. MS, mass spectrometry; SILAC, stable isotope labeling by amino acids in cell culture.

Next, we used a SILAC–MS approach to determine the consequences of Tb927.8.3070 depletion on the mitochondrial proteome. After 3 days of RNAi induction, mitochondria-enriched fractions of uninduced (control) and induced SILAC Tb927.8.3070-RNAi cells were prepared and analyzed by quantitative MS. We found that 14 subunits of the F_o_ moiety of the F_1_F_o_ ATP synthase complex significantly decreased upon Tb927.8.3070 depletion (Fig. 5*A*, *green*), with 10 of these subunits (ATPTB1, ATPTB2, ATPTB5–ATPTB7, ATPTB10–ATPTB13) more than 1.5-fold depleted (Table S2). However, the levels of all six subunits of the F_1_ moiety (α, β, γ, δ, ε, and p18) were unaffected in the same experiment (Fig. 5*A*, *orange*). This is similar to what was observed upon knockdown of the F_o_ subunit ATPTB1 or the peripheral stalk subunits OSCP and ATPTB2 (21, 30, 33), and in contrast to the knockdown of the F_1_ subunits α, β, and p18, where the stability of both F_1_ and F_o_ components was affected (25, 26, 33). There were seven proteins not annotated as ATP synthase subunits found to be significantly more than 1.5-fold depleted in this experiment (Fig. 5*B*): the axonemal inner arm dynein light chain (51), three trypanosomatid-specific proteins of unknown function that are found in the mitochondrial importome (Tb927.6.590, Tb927.2.5930, and Tb927.10.9120), and three trypanosomatid-specific proteins of unknown function not detected in the importome (Tb927.9.7980, Tb927.10.1430, and Tb927.11.9940).

### Depletion of Tb927.8.3070 affects mitochondrial physiology

**Figure 6:**
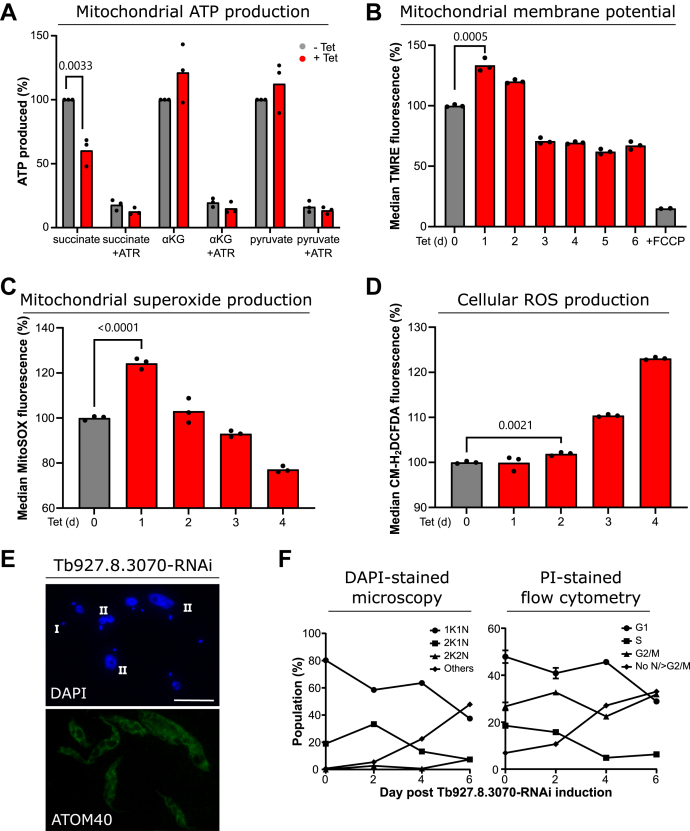
**Tb927.8.3070 knockdown affects mitochondrial physiology.***A*, ATP production analysis of crude mitochondrial lysates from uninduced and 3 days induced Tb927.8.3070-RNAi cells. Oxidative phosphorylation or substrate-level phosphorylation was induced by the addition of either succinate, α-ketoglutarate (α-KG), or pyruvate plus succinate (pyruvate) as substrates. Atractyloside (ATR), an inhibitor of the ATP/ADP translocator, selectively prevents mitochondrial ATP production. Three biological replicates were analyzed. The *p* value was calculated by an unpaired *t* test. The mean of ATP production detected in crude mitochondrial lysates of uninduced cells treated with the respective substrate was set to 100%. *B*, measurement of ΔΨm in the uninduced and induced Tb927.8.3070-RNAi cell line. Analysis was performed by measuring TMRE fluorescence using flow cytometry. Three biological replicates were analyzed per time point. The *p* value was calculated using an unpaired *t* test. The average TMRE fluorescence of the uninduced cell line was set to 100%. Addition of FCCP, a ΔΨm uncoupler, acts as a negative control. *C*, measurement of mitochondrial superoxide production in uninduced and induced Tb927.8.3070-RNAi cell lines. Analysis was performed by measuring MitoSOX fluorescence using flow cytometry. Three biological replicates were analyzed per time point. The *p* value was calculated using an unpaired *t* test. The average MitoSOX fluorescence of the uninduced cell line was set to 100%. *D*, measurement of cellular reactive oxygen species production in the uninduced and induced Tb927.8.3070-RNAi cell line. Analysis was performed by measuring CM-H_2_DCFDA fluorescence using flow cytometry. Three biological replicates were analyzed per time point. The *p* value was calculated using an unpaired *t* test. The average CM-H_2_DCFDA fluorescence of the uninduced cell line was set to 100%. *E*, representative IF image of Tb927.8.3070-RNAi cells induced for 4 days. *Top panel*, DAPI marks both nuclear and mitochondrial DNA. *Bottom panel*, ATOM40 serves as a mitochondrial marker. The scale bar represents 10 μm. *F*, analysis of cell cycle progression upon depletion of Tb927.8.3070 by RNAi. *Left panel*, the visualization of DAPI-stained cells (n > 130 cells per time point). *Right panel*, the quantification of PI fluorescence using flow cytometry. The proportion of cells found in each cell cycle stage are shown as a percentage of the total population. Flow cytometry analysis was performed in three biological replicates. The average percent value here is presented with error bars showing standard deviation. ATOM40, atypical translocase of the outer membrane 40; CM-H_2_DCFHDA, chloromethyl derivative of H_2_DCFHDA; DAPI, 4′,6-diamidino-2-phenylindole; FCCP, carbonyl cyanide-*4*-(trifluoromethoxy)phenylhydrazone; IF, immunofluorescence; PI, propidium iodide; TMRE, tetramethylrhodamine ethyl ester.

Mitochondrial ATP production by oxidative phosphorylation requires both intact F_o_ and F_1_ moieties of the ATP synthase. In order to determine the role of Tb927.8.3070 in this process, we performed *in organello* ATP production assays using digitonin-extracted crude mitochondrial fractions of the uninduced and induced RNAi cell line (52). It had previously been shown that in such assays, depletion of the F_o_ subunit ATPTB1 or the peripheral stalk subunit ATPTB2 strongly impeded ATP production by oxidative phosphorylation using succinate as a substrate (33). When we measured the effect of Tb927.8.3070 depletion on ATP production levels, we found a significant decrease in the succinate-mediated ATP production of around 50% by day 3 postinduction (Fig. 6*A*). This decrease in activity is specific to oxidative phosphorylation, as the level of ATP produced by substrate-level phosphorylation stimulated by either α-ketoglutarate or pyruvate was unaffected. Tb927.8.3070 thus is required for efficient oxidative phosphorylation in mitochondria of procyclic cells.

As in other systems, the F_1_F_o_ ATP synthase of procyclic *T. brucei* utilizes the ΔΨ_m_ generated by the mitochondrial electron transport chain to power ATP production. One known side effect of reduced ATP synthase activity in the procyclic form is the hyperpolarization of the IM, with an overshoot of ΔΨ_m_ to a level higher than that of the steady state, as was seen in cells upon the depletion of F_o_ subunit ATPTB1 or treatment with the F_o_ proton pore inhibitor oligomycin (30, 36, 53).

We therefore measured the ΔΨ_m_ of uninduced and induced Tb927.8.3070-RNAi cells with the ΔΨ_m_-sensitive dye tetramethylrhodamine ethyl ester (TMRE) by flow cytometry (Fig. 6*B*). By day 1 postinduction, there was a significant increase in the ΔΨ_m_, a level that remained high at day 2 postinduction and then decreased by day 3 to a level 75% of that in uninduced cells. The observed transient hyperpolarization in ΔΨ_m_ at day 1 (Fig. 6*B*) can be explained as follows. The initial disruption of the ATP synthase complex prevents the movement of protons through the F_o_ subcomplex. This causes build up of protons in the intermembrane space (IMS) because of continued complex III and IV function, thus increasing the proton ion gradient between the IMS and the matrix. This is expected to increase the production of mitochondrial reactive oxygen species (ROS) (54, 55, 56). We therefore measured the production of mitochondrial superoxide and cellular ROS in Tb927.8.3070-RNAi cells before and after induction using flow cytometry and the ROS-sensitive dyes MitoSOX and chloromethyl derivative of H_2_DCFHDA (CM-H_2_DCFHDA), respectively. Indeed, a significant transient increase in mitochondrial superoxide production by day 1 postinduction (Fig. 6*C*) was observed, which subsequently led to an increase in cellular ROS production by day 2 postinduction (Fig. 6*D*). Mitochondrial superoxide levels immediately peaked and then decreased over time after Tb927.8.3070 depletion, presumably because of the rewiring of electrons from the respiratory complexes contributing to ΔΨ_m_ toward the nonproton pumping alternative oxidase (30, 57, 58, 59). However, cellular ROS molecules continued to accumulate, in a similar fashion to that seen upon the depletion of F_o_ subunit ATPTB1 (30), a culmination of the nonreversible damage caused by Tb927.8.3070 depletion, and likely a direct effector of the growth phenotype.

Finally, we investigated whether the growth retardation caused by the lack of Tb927.8.3070 is accompanied by disturbance of the cell cycle. Analysis of the DNA content of these cells by fluorescence microscopy of 4′,6-diamidino-2-phenylindole-stained cells (Fig. 6, *E* and *F*, *left graph*) and flow cytometry analysis of propidium iodide–stained cells (Fig. 6*F*, *right graph*) showed a defect in cell cycle progression concomitant with the growth phenotype. After day 2 postinduction, a reduction in the proportion of cells going through kinetoplast, or mitochondrial DNA, division (2K1N cells, S stage of the cell cycle, *square symbols* in Fig. 6*F*) was seen, with a large decrease in the proportion of cells beginning the cell cycle seen between days 4 and 6 (1K1N, G1, *circle symbols* in Fig. 6*F*). There was also an increase in the proportion of zoid cells with no nucleus (marked with I in Fig. 6*E*, *diamond symbols* in Fig. 6*F*, incorporated into the “others” category in Fig. 6*F*, *left panel*), and cells with large or multiple nuclei (marked with II in Fig. 5*E*, *diamond symbols* in Fig. 6*F*, >G2/M and incorporated in the “others” category). Moreover, across this time course, approximately 50% of all cells had an aberrant DNA content by day 6. Thus, Tb927.8.3070 is required for normal growth and cell cycle progression in procyclic *T. brucei*. However, there are no massive changes in mitochondrial morphology after depletion of Tb927.8.3070 in induced cells (Fig. 6*E*, *bottom panel*).

## Discussion

The *T. brucei* F_1_F_o_ ATP synthase peripheral stalk subunit *b* could not be identified by sequence homology. This is not that surprising since closely related organism *E. gracilis*, which has an F_1_F_o_ ATP synthase with similarity in architecture and subunit composition to the *T. brucei* version (13, 23, 33, 34), has a highly diverged subunit *b* (14). The peripheral stalk of the *E. gracilis* complex has an extended OSCP, which unusually interacts with the central stalk subunit γ, and with a divergent extended subunit *d*, which, unlike its opisthokont counterpart, has a TMD (21, 60). The euglenozoan-specific ATPTB3 and ATPTB4 stabilize the interaction between subunit *d* and OSCP and facilitate an interaction with the catalytic core of the F_1_ moiety *via* one copy of p18 (13). *E. gracilis* subunit *b* could not be detected by sequence homology but was identified based on its structural similarity, position, and topology within the structure. It contains one TMD and is truncated in comparison to its yeast counterpart. Moreover, unlike subunit *b* in other complexes, it does not interact directly with OSCP, the dimerization interface, or subunit 8. Instead, it interacts extensively with subunit *d* along the external peripheral stalk and with subunits *a* and *f* in the membrane-embedded region.

Interestingly, the *T. brucei* F_1_F_o_ ATP synthase has an extended OSCP as well as a highly diverged extended subunit *d* similar to that of *E. gracilis*. Based on the structure of the peripheral stalk of *E. gracilis* and because no subunit *b* could be identified in *T. brucei*, the question of whether the trypanosomal version of the protein could be either further reduced in length or even be completely absent was raised (14).

We have identified a 17 kDa mitochondrial IM protein, essential for normal growth of procyclic *T. brucei*, which contains a single predicted TMD close to the N terminus. The tagged protein pulls down essentially all known F_1_F_o_ ATP synthase subunits and comigrates with the monomer of the F_1_F_o_ ATP synthase when analyzed by BN-PAGE. Knockdown of the protein preferentially reduces the abundance of F_o_ ATP synthase subunits but not F_1_ subunits, and results in a decrease of the F_o_ moiety–containing complexes on BN gels. RNAi analysis shows that the protein is involved in oxidative phosphorylation and ΔΨ_m_ maintenance in a similar way to that previously described for other trypanosomal F_o_ ATP synthase subunits. Finally, HHpred analysis reveals structural similarities to F_1_F_o_ ATP synthase subunit *b* of other species. These results indicate that the 17 kDa protein may be the so far elusive subunit *b* of the peripheral stalk of the F_1_F_o_ ATP synthase of trypanosomes. Should this be the case, the highly diverged subunit *b*, while truncated, is 30% larger than the *E. gracilis* protein (145 amino acids compared with 112 amino acids). This suggests that the *T. brucei* ATP synthase peripheral stalk may have a similar global architecture than the one in *E. gracilis*, although the longer putative trypanosomal subunit *b* could potentially show unique subunit interactions. Alternatively, it also possible that Tb927.8.3070 is a late assembly factor that is not present in the fully assembled F_1_F_o_ ATP synthase dimer. However, should this be the case, no similarity with subunit *b* from *Euglena* or from other organisms would be expected.

Interestingly, Tb927.8.3070 has not been detected in previous pull-down MS analyses of the *T. brucei* F_1_F_o_ complexes (33, 61). This could be explained by complex disruption during extraction and/or by its low molecular mass and having few tryptic cleavage sites, which may make detection by proteomic methods difficult (62). However, the putative *T. brucei* subunit *b* had previously been detected in a pull-down analysis of the mitochondrial calcium uniporter, along with 19 other subunits of the ATP synthase (63). Should Tb927.8.3070 be an assembly factor instead of a highly diverged subunit *b*, its pull down might have precipitated a late F_1_F_o_ ATP synthase assembly intermediate that otherwise would be difficult to identify.

Depletion of Tb927.8.3070 led to a more than 1.5-fold decrease in abundance of nine F_o_ and peripheral stalk subunits, showing that despite its small size and reduced subunit interactions, it is an essential subunit for F_o_ and peripheral stalk assembly and/or for maintenance of their structural integrity. In contrast, all F_1_ subunits remain stable even in the absence of Tb927.8.3070, similar to what has been shown in yeast (64, 65, 66), where the F_1_ moiety assembles independently of an intact F_o_ moiety.

Besides many previously identified F_1_F_o_ ATP synthase subunits, our SILAC pull-down and SILAC RNAi analyses recovered a number of trypanosome-specific proteins of unknown function. We analyzed all nine kinetoplastid-specific proteins of unknown function that were found to be either more than 10-fold enriched in the Tb927.8.3070 SILAC pull down (Fig. S1) and/or more than 1.5-fold downregulated in the Tb927.8.3070 SILAC RNAi experiments (Fig. 5*B*) in more detail (Table S3). Interestingly, for two of these proteins, HHpred found convincing similarities with F_o_ ATP synthase subunits of other organisms. Tb927.6.590 (12.3 kDa), a mitochondrial IM protein (41, 42), was 2.2-fold downregulated in level after ablation of Tb927.8.3070 and was detected as an interactor of the trypanosomal Ca^2+^ uniporter (63)*.* HHpred predictions suggested that Tb927.6.590 might be a trypanosomal ortholog of ATPEG3, which in *Euglena* together with ATPTB1, ATPTB6, ATPTB12, and ATPEG5, 6, 8 forms a phylum-specific subcomplex that tightly interacts with the conserved core of the F_o_ ATP synthase moiety (13). Tb927.4.1760 (14.4 kDa), another mitochondrial IM protein (41, 42), was found 11.4-fold enriched in the Tb927.8.3070 pull down. HHpred detected a similarity to predominantly the thioredoxin-like domain of the F_o_ ATP synthase subunit ATPTG4 of *Toxoplasma gondii* (16)*.* Tb927.4.1760 belongs to a group of proteins whose level is reduced upon depletion of cardiolipin in procyclic *T. brucei*, which is why it was termed cardiolipin-dependent protein 17 (46). Interestingly, ATPTG4 contributes to the binding of one of 15 cardiolipins that were detected in the structure of the F_o_ ATP synthase moiety of the *Toxoplasma* F_1_F_o_ ATP synthase (16). These cardiolipins mediate a stabilization network of interaction in the membrane region of the F_o_ ATP synthase moiety, as is the case in the ATP synthase complexes of other organisms (67, 68, 69, 70), supporting the idea that Tb927.4.1760 might be a trypanosomal ATPTG4 ortholog. Cardiolipin may stabilize the F_1_F_o_ ATP synthase in procyclic *T. brucei* as had been shown for bloodstream forms (71). Additionally, Tb927.9.7980, which is downregulated twofold after RNAi of Tb927.8.3070, also shows low similarity with various ATP synthases from different organisms. However, the overlap of similarity was very small, and Tb927.9.7980 was also suggested to be similar to the yeast IMS protein Mic17. Thus, its function remains unknown. For the remaining hypothetical proteins, HHpred revealed neither similarity to F_1_F_o_ ATP synthase subunits nor convincing similarities to other known proteins. Nevertheless, it is still possible that some of these proteins are yet unknown trypanosome-specific subunits of the F_1_F_o_ ATP synthase. Good candidates would be Tb927.2.5930 and Tb927.2.5140 as they are both efficiently coimmunoprecipitated with Tb927.8.3070 and reduced in level after its depletion (Table S3), in a similar fashion to *T. brucei* F_o_ and peripheral stalk subunits (Figs. 5 and S1).

Knockdown of Tb927.8.3070 results in physiological changes that already have been described for other F_o_ ATP synthase subunits. Interestingly, we also observed a cell cycle phenotype. Kinetoplast DNA replication seems to be inhibited, and possibly as a consequence the whole cell cycle is disturbed; this cell cycle phenotype is in agreement with an RNAi analysis of ATPTB2/subunit *d* (72). We did not find any published results reporting the same type of analysis for any other F_1_ or F_o_ ATP synthase subunits of trypanosomes. This suggests that knockdown of another subunit of the F_1_F_o_ ATP synthase could cause the same phenotype. Indeed, a recent systematic analysis of RNAi cell lines targeting 101 randomly chosen mitochondrial proteins of unknown function in procyclic *T. brucei* showed that knockdown of approximately a third of these proteins caused a growth retardation concomitant with abnormalities in cell cycle progression (72). This suggests that an abnormal cell cycle is a common phenotype observed after depletion of mitochondrial proteins that are required for normal growth. However, in the Tb927.8.3070-RNAi cell line, the fraction of cells with abnormal N/K configurations was >40%, whereas Mbang-Benet *et al*. (72) reported such strong phenotypes for only three of 37 cell lines showing cell cycle abnormalities. Presently, we do not know the underlying mechanism that causes the cell cycle phenotype detailed here.

While the present study was under revision, the structure of the F_1_F_o_ ATP synthase dimer of *T. brucei* was reported (73), which surprisingly appears to contain a subunit *b* homolog that is different from Tb927.8.3070. This seems to favor the interpretation that Tb927.8.3070 is a late assembly factor rather than being subunit *b*; however, it cannot be excluded that trypanosomal F_1_F_o_ ATP synthase complexes might be heterogenous and contain different subunit *b*-like proteins. Further work is required to resolve this issue.

## Experimental procedures

### Transgenic cell lines

Transgenic *T. brucei* cell lines were generated using the procyclic strain 29-13 (74). Procyclic forms were cultivated at 27 °C in SDM-79 (75) supplemented with 10% (v/v) fetal calf serum containing G418 (15 μg/ml), hygromycin (25 μg/ml), puromycin (2 μg/ml), and blasticidin (10 μg/ml) as required. RNAi or protein expression was induced in cell lines by adding 1 μg/ml tetracycline to the medium.

The Tb927.8.3070-RNAi cell lines were prepared using a pLew100-derived vector containing a blasticidin resistance gene, with the generation of a stem–loop construct occurring by the insertion of the RNAi inserts in opposing directions. The loop is formed by a 460 bp spacer fragment. RNAi plasmids were prepared targeting Tb927.8.3070 *via* its entire ORF or its entire 3′ UTR (as designated on TriTrypDB). PCR was used to amplify the RNAi targets with primers (F) ACATTAAAGCTTGGATCCATGGCCTATGTTTCTCCAGC and (R) CGTATTTCTAGACTCGAGCTACTTCGGTAACCGCTGCT (ORF), and (F) ACATTAAAGCTTGGATCCCGGCGGTGCGTGGTTG and (R) CGTATTTCTAGACT CGAGAACGAGAGGAGAGAGACCGC (3′ UTR). RNAi efficiency was verified by RNA extraction and Northern blot, as detailed (76).

To produce the plasmid for ectopic expression of C-terminal triple c-myc-tagged Tb927.8.3070, the complete ORF was amplified by PCR using primers (F) ACATTAAAGCTTATGGCCTATGTTTCTCCAGC and (R) CGTATTGGATCCCTTCGGTAACCGCTGCTGAT. The PCR product was cloned into a modified pLew100 vector, which contains a puromycin resistance gene as well as a triple epitope tag. Protein expression was verified by SDS-PAGE and immunoblotting of cell lysates.

### Subcellular localization

The subcellular localization of Tb927.8.3070 was analyzed by generating crude mitochondrial-enriched fractions. 1 × 10^8^ cells were incubated in 0.6 M sorbitol, 20 mM Tris–HCl (pH 7.5), 2 mM EDTA, and pH 8 containing 0.015% (w/v) digitonin on ice for 10 min to solubilize the cell membranes. Centrifugation for 5 min at 6800*g* at 4 °C yielded a supernatant that is enriched for cytosolic proteins and a crude mitochondrial extract pellet. 2 × 10^6^ cell equivalents of each fraction were analyzed by SDS-PAGE and Western blotting.

The mitochondria-enriched pellet was resuspended in 100 mM Na_2_CO_3_ (pH 11.5), incubated on ice for 10 min, and centrifuged for 10 min at 100,000*g*, 4 °C to differentiate soluble or loosely membrane-associated proteins from integral membrane proteins. 2 × 10^6^ cell equivalents of each fraction were analyzed by SDS-PAGE and Western blotting.

Commercially available antibodies were used as follows: mouse c-Myc (Invitrogen; 1:2000 dilution) and mouse elongation factor 1-alpha (Merck Millipore; 1:10,000 dilution). The polyclonal VDAC (1:1000 dilution) (77) and cytochrome *c* (1:100 dilution) (42) antibodies previously produced in our laboratory were also used. Secondary antibodies for immunoblot analysis were IRDye 680LT goat antimouse and IRDye 800CW goat anti-rabbit (both LI-COR Biosciences; 1:20,000 dilution).

For IF analysis, cells were fixed with 4% paraformaldehyde in PBS, permeabilized with 0.2% Triton X-100 in PBS, and blocked with 2% bovine serum albumin (BSA). Primary antibodies used were mouse anti-c-Myc (1:50 dilution) and rabbit anti–atypical translocase of the outer membrane 40 (1:1000 dilution), and secondary antibodies were goat antimouse Alexa Fluor 596 and goat anti-rabbit Alexa Fluor 488 (both Thermo Fisher Scientific; 1:1000 dilution).

Slides were mounted with VectaShield containing 4′,6-diamidino-2-phenylindole (Vector Laboratories). Images were acquired with a DFC360 FX monochrome camera (Leica Microsystems) mounted on a DMI6000B microscope (Leica Microsystems). Images were analyzed using LAS AF software (Leica Microsystems) and ImageJ (NIH).

### BN-PAGE and quantification

Mitochondrial-enriched pellets from 1 × 10^8^ cells/sample were incubated for 15 min on ice in 20 mM Tris–HCl (pH 7.4), 50 mM NaCl, 10% glycerol, 0.1 mM EDTA, and 1 mM PMSF containing 1% (w/v) digitonin to solubilize mitochondrial membranes. After centrifugation for 15 min at 20,817*g*, 4 °C, the supernatants were separated on 4 to 13% gradient gels. The gel was then incubated in SDS-PAGE running buffer (25 mM Tris, 1 mM EDTA, 190 mM glycine, and 0.05% [w/v] SDS) to aid the transfer of proteins to the membrane. Quantification of bands was performed using ImageJ.

### ATP production assay

ATP production was measured using substrates succinate, pyruvate, and α-ketoglutarate as described (52). The ADP/ATP carrier inhibitor atractyloside was added to samples as a negative control. ATP Bioluminescence assay kit CLS II (Roche Applied Science) was used to measure the ATP concentration of the samples in a luminometer plate reader.

### Flow cytometry

The TMRE Mitochondrial Membrane Potential kit (Abcam) was used to measure ΔΨm. 1 × 10^6^ cells from each sample were resuspended in 1 ml media. All samples were preincubated with or without 20 μM uncoupler carbonyl cyanide-*4*-(trifluoromethoxy)phenylhydrazone to provide a negative control for 10 min at 27 °C, supplemented with 100 nM TMRE, and left at 27 °C for 20 min. Cells were pelleted at 2000*g* for 5 min in 5 ml polystyrene round bottom tubes (BD Falcon; catalog no.: 352052), and washed three times in 5 ml 0.2% BSA in PBS-glucose solution containing 6 mM glucose. Cell pellets were resuspended in 500 μl 0.2% BSA in PBS-glucose solution, left for 30 min in foil, and analysis was performed on Novocyte instrument (Agilent), using the 488 nm laser for excitation and detection using the B586/20 nm filter. Data were analyzed using FlowJo (Becton Dickinson).

For the measurement of ROS production, 3 × 10^6^ cells/sample were analyzed. The same protocol as aforementioned was used, but cells were supplemented with either 5 μM MitoSOX (Thermo Fisher Scientific) or 10 μM CM-H_2_DCFDA (Thermo Fisher Scientific). The 488 nm laser was used for excitation, with detection using the B586/20 nm filter for MitoSOX and the B530/30 nm filter for CM-H_2_DCFDA.

For cell cycle analysis, 1 × 10^6^ cells/sample were fixed with 70% ice-cold methanol for 10 min, washed in PBS twice, and stained with 10 μg/ml propidium iodide plus 10 μg/ml RNase A. After incubation for 45 min, the samples were analyzed using the 488 nm laser for excitation and detection using the B586/20 nm filter.

### SILAC-based proteomics and IP experiments

Cells were washed in PBS and taken up in SDM-80 supplemented with 5.55 mM glucose, either light (^12^C_6_/^14^N_χ_) or heavy (^13^C_6_/^15^N_χ_) isotopes of arginine (1.1 mM) and lysine (0.4 mM) (Euroisotop), and 10% dialyzed fetal calf serum (BioConcept). To guarantee complete labeling of all proteins with heavy amino acids, the cells were cultured in SILAC medium for 6 to 10 doubling times.

The Tb927.8.3070-RNAi cell line was induced with tetracycline for 3 days. About 1 × 10^8^ uninduced and 1 × 10^8^ induced cells were harvested and mixed. Crude mitochondria-enriched pellets were obtained by incubating 2 × 10^8^ cells on ice for 10 min in 0.6 M sorbitol, 20 mM Tris–HCl (pH 7.5), and 2 mM EDTA (pH 8) containing 0.015% (w/v) digitonin, and centrifugation (5 min/6800*g*/4 °C). The digitonin-extracted mitochondria-enriched pellets generated from these mixed cells were then analyzed.

For SILAC-IP experiments, cells were induced for 1 day. About 1 × 10^8^ uninduced and 1 × 10^8^ induced cells were harvested in triplicate, mixed, and subjected to a co-IP protocol as follows.

About 2 × 10^8^ cells were solubilized for 15 min on ice in 20 mM Tris–HCl (pH 7.4), 0.1 mM EDTA, 100 mM NaCl, 25 mM KCl containing 1% (w/v) digitonin, and 1× Protease Inhibitor mix (Roche; free of EDTA). After centrifugation (15 min, 20,000*g*, 4 °C), the lysate (IN) was transferred to 50 μl of bead slurry, which had been previously equilibrated with respective lysis buffer. The bead slurries used were c-myc-conjugated (EZview red rabbit anti-c-myc affinity gel; Sigma). After incubating at 4 °C for 2 h, the supernatant containing the unbound proteins was removed, the bead slurry was washed three times with lysis buffer, and the bound proteins were eluted by boiling the resin for 10 min in 2% SDS in 60 mM Tris–HCl (pH 6.8) (IP). Pull down of the bait was confirmed by SDS-PAGE and Western blotting, with 5% of both the input and the flow-through samples and 50% of the IP sample loaded.

All SILAC experiments were performed in three biological replicates including a label switch and analyzed by LC–MS.

### LC–MS and data analysis

Samples generated in Tb927.8.3070 SILAC RNAi experiments (n = 3) were processed for LC–MS analysis (including reduction and alkylation of cysteine residues, tryptic in-solution digestion) as described before (41). Eluates of Tb927.8.3070 SILAC-IP experiments (n = 3) were loaded onto an SDS gel, and electrophoresis was performed until the proteins had migrated into the gel for approximately 1 cm. Proteins were visualized using colloidal Coomassie blue, protein-containing parts of the gel were excised en bloc, and cut into smaller cubes, followed by reduction and alkylation of cysteine residues and tryptic in-gel digestion as described before (41).

LC–MS analyses of tryptic peptide mixtures were performed using an Orbitrap Elite mass spectrometer (Thermo Fisher Scientific) connected to an UltiMate 3000 RSLCnano HPLC system (Thermo Fisher Scientific). Peptides were loaded and concentrated on nanoEase M/Z Symmetry C18 precolumns (20 mm × 0.18 mm; flow rate, 10 μl/min; Waters) and separated using a nanoEase M/Z HSS C18 T3 analytical column (250 mm × 75 μm; particle size, 1.8 μm; packing density, 100 Å; flow rate, 300 nl/min; Waters). A binary solvent system consisting of 0.1% formic acid (solvent A) and 30% acetonitrile/50% methanol/0.1% formic acid (solvent B) was used. Peptides of all experiments were loaded and concentrated for 5 min at 7% B, followed by peptide elution applying the following gradients: 7 to 60% B in 295 min, 60 to 95% B in 35 min, and 5 min at 95% B (SILAC RNAi) or 7 to 50% B in 105 min, 50 to 95% B in 45 min, and 5 min at 95% B (SILAC–IP experiments).

Mass spectrometric data were acquired in data-dependent mode applying the following parameters: mass range of *m/z* 370 to 1700, resolution of 120,000 at *m/z* 400, target value of 1 × 10^6^ ions, and maximum injection time of 200 ms for MS survey scans. A TOP25 method was used for low-energy collision-induced dissociation of multiply charged peptides in the linear ion trap at a normalized collision energy of 35%, an activation *q* of 0.25, an activation time of 10 ms, a target value of 5000 ions, a maximum injection time of 150 ms, and a dynamic exclusion time of 45 s.

MaxQuant/Andromeda (version 1.6.5.0, (78, 79)) was used for protein identification and SILAC-based relative quantification (80). Database searches were performed using the proteome of *T. brucei* TREU927 downloaded from the TriTryp database (version 8.1; containing 11,067 entries) and a list of common contaminants provided by MaxQuant. Data were processed using tryptic specificity with a maximum number of two missed cleavages and mass tolerances of 4.5 ppm for precursor and 0.5 Da for fragment ions. Carbamidomethylation of cysteine residues was set as fixed modification, N-terminal acetylation and oxidation of methionine were considered as variable modifications, and Arg10 and Lys8 were set as heavy labels. For protein identification, at least one unique peptide with a minimum length of seven amino acids was required. A false discovery rate of 1%, calculated as described before, was applied at the level of both peptide spectrum matches and protein identifications. The options “requantify” and “match between runs” were enabled. SILAC ratios were calculated based on unique peptides and at least one ratio count. The mean log_10_ (SILAC–IPs) or mean log_2_ (SILAC RNAi) of normalized ratios (with/without Tet-induced) was calculated, and a one-sided (SILAC–IP data) or two-sided (SILAC RNAi data) Student’s *t* test was performed to determine *p* values. Results of protein identification and quantification are provided in the Supporting Information as Table S1 (Tb927.8.3070 SILAC–IP data) and S2 (Tb927.8.3070 SILAC RNAi data).

## Data availability

The MS data have been deposited to the ProteomeXchange Consortium (81) *via* the PRIDE (82) partner repository and are accessible using the dataset identifiers PXD031256 (SILAC–IP data) and PXD031259 (SILAC RNAi data). Annotated spectra of identified peptides can be inspected at MSViewer (83) using the search keys iyg4zvn2ww (SILAC–IP data) and 0qpdxuwckb (SILAC RNAi data).

## Supporting information

This article contains supporting information (41, 42, 46, 63, 84, 85).

## Conflict of interest

The authors declare that they have no conflicts of interest with the contents of this article.
