# Supplementary material for: Characterization of a highly diverged mitochondrial ATP synthase Fo subunit in Trypanosoma brucei
Source: J Biol Chem. 2022 Mar 12;298(4):101829. doi: 10.1016/j.jbc.2022.101829 (PMC9034290; doi:10.1016/j.jbc.2022.101829)
Supplement: Supplemental Figures S1 and S2, Tables S1–S3 [file mmc3.pdf]

Characterization of a highly diverged mitochondrial ATP synthase F_o subunit in *Trypanosoma brucei*

Caroline E. Dewar[1)], Silke Oeljeklaus[2)], Christoph Wenger[1)] Bettina Warscheid[2),3)] *

and André Schneider[1)]*

Running title: Highly diverged F_o subunit of *T. brucei*

[1]Department of Chemistry, Biochemistry and Pharmaceutical Sciences, University of Bern, Freiestrasse 3, Bern CH-3012, Switzerland

[2)] Department of Biochemistry, Theodor Boveri-Institute, University of Würzburg, 97074 Würzburg, Germany

[3)] CIBSS Centre for Integrative Biological Signalling Studies, University of Freiburg, 79104 Freiburg, Germany

* To whom correspondence should be addressed: bettina.warscheid@biologie.uni-freiburg.de, andre.schneider@unibe.ch

Keywords: ATP synthase, mitochondria, Trypanosoma brucei, proteomics, protozoan

Supporting Information:

Figure S1: Further proteins that interact with Tb927.8.3070

Figure S2: *In silico* analysis of Tb927.8.3070

Table S1: Tb927.8.3070 SILAC-IP data

Table S2: Tb927.8.3070 SILAC RNAi data

Table S3: List of proteins found more than 5-fold enriched in the Tb927.8.3070-myc SILAC CoIP and those found more than 1.5-fold downregulated in Tb927.8.3070 SILAC RNAi experiment.

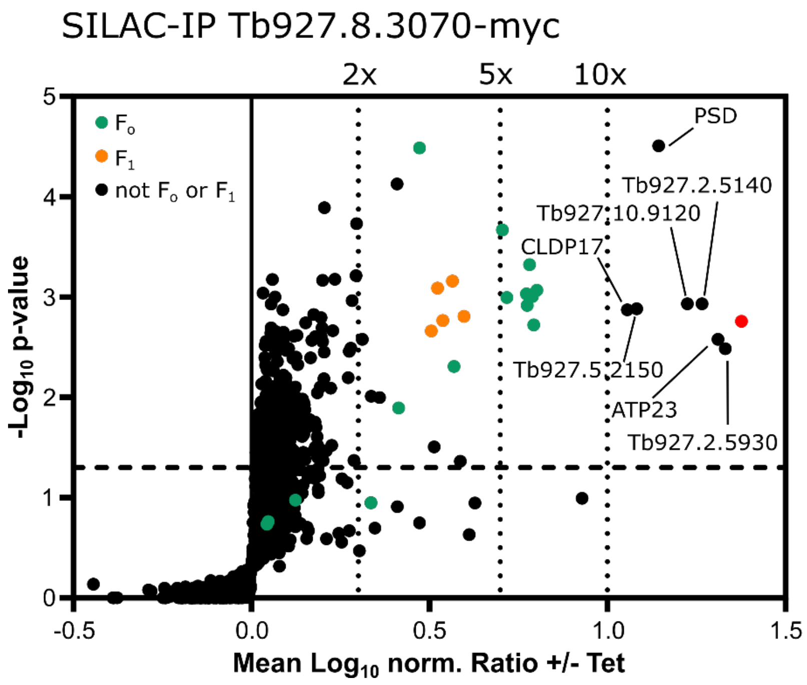

Fig. S1. Further proteins that interact with Tb927.8.3070

A volcano plot depicting of the SILAC-IP analysis of crude mitochondrial extracts from Tb927.8.3070-myc expressing cells as shown in Fig 1C. Proteins more than 10-fold enriched are labelled with either their name or accession numbers.

A

No	Hit	Prob	E-value	P-value	Score	SS	Cols	Query HMM	Template HMM
1	4OD8_D DNA polymerase processi	51.6	5.1	9.2E-05	27.9	-0.3	11	9-19	37-47 (52)
2	6RD5_0 ASA-10: Polytomella F-A	51.1	8.4	0.00015	29.0	0.7	41	40-82	6-46 (82)
3	5CEN_A Mitogen-activated prote	48.9	94	0.0017	24.3	6.2	88	56-144	185-289 (300)
4	6R8F_D BRISC complex subunit A	46.1	50	0.00091	27.6	4.6	41	103-145	215-255 (267)
5	6ICR_D Coronin-like protein; C	45.3	1E+02	0.0018	21.5	5.3	32	109-140	6-38 (53)
6	2O6N_A RH4B designed peptide;	43.7	58	0.001	21.1	3.6	22	101-131	12-33 (35)
7	5CX2_B Coronin; 4 helix bundle	41.7	1.2E+02	0.0022	20.8	5.1	32	109-140	2-34 (49)
8	1Z0K_D FYVE-finger-containing	40.2	1.4E+02	0.0026	20.7	5.9	68	71-145	2-69 (69)
9	7KAM_D Protein transport prote	36.8	3.9E+02	0.007	24.7	9.2	125	7-142	168-303 (719)
10	5X6O_G DNA damage checkpoint p	34.8	96	0.0017	29.7	5.1	43	103-145	76-125 (747)
11	6KPG_R Cannabinoid receptor 1;	34.2	1.9E+02	0.0033	23.1	5.8	43	103-145	5-47 (469)
12	6K15_E High temperature lethal	33.9	1.9E+02	0.0034	21.7	5.5	48	95-142	20-73 (78)
13	6FKF_b ATP synthase subunit b,	33.4	2.1E+02	0.0038	20.6	6.7	96	45-140	21-116 (184)
14	3M91_A Proteasome-associated A	33.2	89	0.0016	20.4	3.4	22	122-143	30-51 (51)
15	6B8H_b ATP synthase subunit 4,	33.1	1.7E+02	0.0031	22.7	5.5	101	40-140	44-148 (209)
16	2MRL_A Uncharacterized protein	32.7	33	0.0006	24.1	1.4	35	103-138	14-48 (72)
17	4P9T_D Catenin alpha-2; Cytosk	30.9	3.3E+02	0.0059	22.1	8.6	64	82-145	103-169 (263)
18	2FU2_A Hypothetical protein SP	30.6	79	0.0014	21.9	3.0	41	105-145	1-41 (102)
19	1DOV_A ALPHA-CATENIN; four-hel	30.5	2.7E+02	0.0048	21.0	8.4	64	82-145	21-87 (181)
20	6SO5_D Tail-anchored protein i	29.6	3E+02	0.0053	21.2	7.1	85	49-144	8-92 (184)
21	4NEU_B Receptor-interacting se	28.6	2.6E+02	0.0047	20.2	8.6	89	56-144	223-330 (333)
22	6LP3_F GTPase-activating prote	27.4	1.1E+02	0.002	23.4	3.5	25	121-145	60-84 (99)
23	6H3C_F BRISC complex subunit A	27.3	1.7E+02	0.0031	27.3	5.3	44	102-145	229-274 (434)
24	2N9B_A Unconventional myosin-X	26.9	1.4E+02	0.0026	20.9	3.7	27	114-140	38-69 (69)
25	6W6L_6 Calcium load-activated	26.6	2E+02	0.0036	21.7	4.7	41	103-143	41-81 (188)
26	6X4S_A Calcium uniporter prote	26.3	78	0.0014	26.5	2.7	37	109-145	22-63 (243)
27	5XA5_A Alpha-catenin-like prot	25.8	4.3E+02	0.0077	21.8	8.1	64	82-145	101-167 (279)
28	5CW4_D Protein FAM175B; Metal	25.7	1.9E+02	0.0033	24.6	4.8	41	103-145	231-271 (289)
29	6ZJ8_F Virulence sensor protei	25.6	1.3E+02	0.0023	17.6	2.9	26	120-145	137-162 (162)
30	3CK4_K GCN4 leucine zipper; Co	25.5	1.3E+02	0.0023	18.9	2.9	20	121-140	14-33 (34)
31	7JGA_b ATP synthase subunit b;	25.4	3.1E+02	0.0056	20.0	6.3	96	45-141	25-125 (170)
32	2B1F_B General control protein	25.2	1.3E+02	0.0024	18.9	2.9	20	121-140	14-33 (34)
33	4JYK_A HTH-type transcriptiona	24.1	2.6E+02	0.0046	18.6	4.8	45	46-91	161-205 (212)
34	6F7B_A Mitotic checkpoint seri	23.7	4.2E+02	0.0076	20.9	9.2	84	56-145	267-360 (361)
35	6CMO_R chimera protein of Solu	23.4	2.7E+02	0.0048	22.7	5.1	43	103-145	39-81 (463)
36	3W92_B Thioester coiled coil p	23.2	1.3E+02	0.0024	18.7	2.7	20	121-140	13-32 (32)
37	1DOW_A ALPHA-CATENIN; four-hel	23.1	4.1E+02	0.0074	20.6	8.4	64	82-145	46-112 (205)
38	1VCS_A Vesicle transport throu	23.0	2.4E+02	0.0044	19.4	4.3	48	82-143	48-95 (102)
39	6YSL_D Motility protein A; Sta	22.8	1.2E+02	0.0021	23.8	3.0	83	47-130	187-269 (270)
40	3RF3_B Vinculin; alpha-helix b	22.6	4.3E+02	0.0078	20.7	8.0	64	82-145	86-152 (258)
41	6CFZ_J Spc34; DASH, Dam1 compl	22.5	1.3E+02	0.0024	25.2	3.4	22	121-142	218-239 (245)
42	5Y3T_A RanBP-type and C3HC4-ty	21.6	2.2E+02	0.0039	18.5	3.6	32	105-136	5-36 (140)
43	6M0Q_J Uncharacterized protein	21.0	3E+02	0.0054	21.3	4.7	47	49-97	2-48 (91)
44	1JOC_A Early Endosomal Autoant	20.7	2.5E+02	0.0044	17.2	4.1	27	116-142	12-38 (125)
45	6N2Y_b2 ATP synthase subunit b	20.3	2.9E+02	0.0051	20.2	4.4	84	45-128	5-93 (168)

B

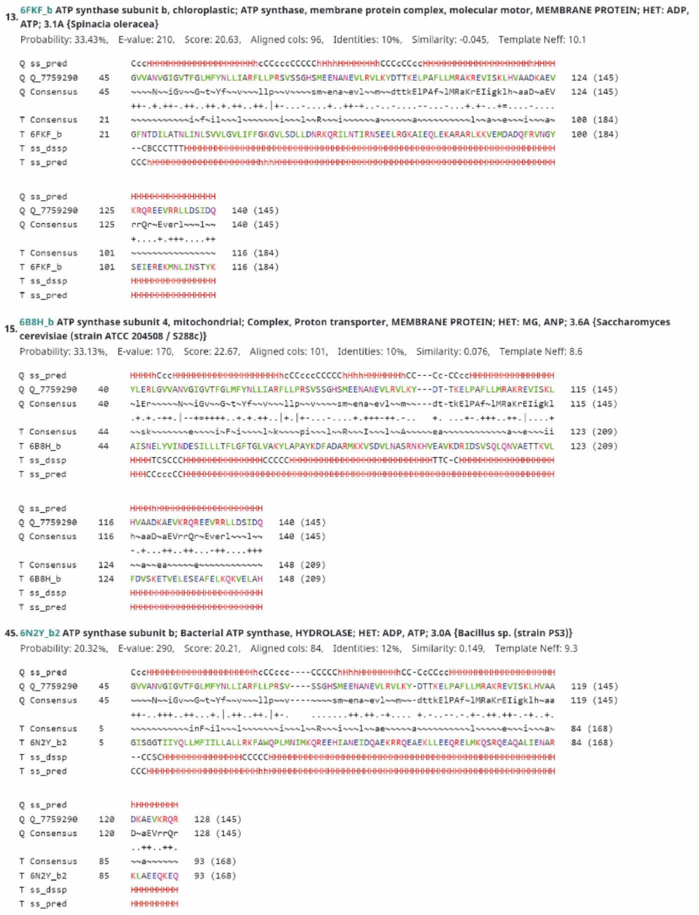

C

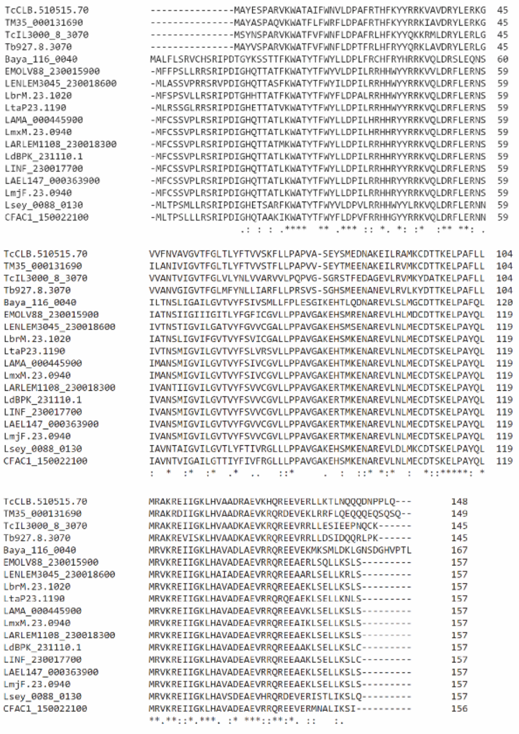

Fig. S2. ***In silico*** **analysis of Tb927.8.3070**

(A) List of HHpred results using Tb927.8.3070 as the input sequence. The hits related to ATP synthase subunit *b* are highlighted in blue. **(B)** The sequence of Tb927.8.3070 that displays secondary structure homology to regions in the ATP synthase subunit *b* of spinach (*Spinacia oleracea*) chloroplasts, yeast (*S. cerevisiae*) and *Bacillus* species using HHpred. **(C)** Sequence alignment between Tb927.8.3070 and its orthologs in Kinetoplastid species using Clustal Omega (84). TcCLB *T. cruzi*, TM *T. theileri*, TcIL *T. congolense*, Baya *B. ayalai*, EMOLV *E. monterogeii*, LENLEM *L. enriettii*, Lbr *L. braziliensis*, Lta *L. tarentolae*, LAMA *L. amazonensis*, Lmx *L. mexicana*, LARLEM *L. arabica*, Ld *L. donovani*, LINF *L. infantum*, LAEL *L. aethiopica*, Lmj *L. major*, Lsey *L. seymouri*, CFAC *C. fasciculata*.

Table S3. List of proteins found more than 5-fold enriched in the Tb927.8.3070-myc SILAC CoIP and those found more than 1.5-fold downregulated in Tb927.8.3070 SILAC RNAi experiment.

ORF and TritrypDB annotation[1]	MW (kDa)	Predicted TMD[2]	Tryptag localisation[3]	Enrichment in Tb927.8.3070 -myc SILAC CoIP[4]	Downregulation in Tb927.8.3070 SILAC RNAi[4]	Associated with MCU[5] [63]	Importome[6] [41]	IM protein[7] [42]	PSI-BLAST[8]	HHpred hit[9]
**Tb927.2.5930**	60.5	N	Mito	21.4x	1.73x	Y	Y	Y	-	Sec31 *S. cerevisiae* (#1) (137/1273AA) Probability 64.08%, p-value 0.0009
**Tb927.2.5140**	17.4	Y	Mito	18.4x	1.48x	Y	Y	nd	-	Sestrin *H. sapiens* (#1) (26/415AA) Probability 34.92%, p-value 0.0013
**Tb927.10.9120**	27.3	N	Mito	16.8x	1.61x	nd	Y	nd	-	Acetaldehyde dehydrogenase *Pseudomonas sp.* (#1) (84/312AA) Probability 48.0%, p-value 0.00015
**Tb927.5.2150**	60.1	N	Mito	12.1x	1.07x	nd	Y	Y	-	Cyt *c* biogenesis protein *B. fragilis* (#1) (41/172AA) Probability 90.11%, p-value 1.5E-05
**Tb927.4.1760** CLDP17 [46]	17.4	N	Mito	11.4x	1.04x	nd	Y	Y	-	ATPTG4 *T. gondii* (#14) (130/267AA) Probability 99.5%, p-value 2E-17
**Tb927.6.590**	12.3	N	Mito	nd	2.23x	Y	Y	Y	-	ATPEG3 *E. gracilis* (#1) (76/116AA) Probability 97.3%, p-value 1.6E-09
**Tb927.9.7980**	15.8	N	Non mito	nd	2.01x	nd	nd	nd	Mix17/ Mic17	ATPTG9 *T. gondii* (#7) (35/166AA) Probability 97.3%, p-value 1.6E-09<br>ATPEG5 *E. gracilis* (#22) (30/90AA) Probability 66.7%, p-value 0.00022<br>ATPTG7 *T. gondii* (#26) (35/236AA) Probability 52.3%, p-value 0.00032<br>ATPTG8 *T. gondii* (#30) (41/208AA) Probability 47.0%, p-value 0.00051<br>ATPTT5 *Tetrahymena thermophila* (#32) (29/273AA) Probability 38.1%, p-value 0.001
**Tb927.11.9940**	20.6	N	na	nd	1.69x	nd	nd	nd	-	Autophagy related protein 16 *H. sapiens* (#1) (125/301) Probability 82.66%, p-value 0.00037
**Tb927.10.1430**	27.3	N	na	nd	1.67x	nd	nd	N	-	Elongation factor P *N. meningitidis* (#1) (44/70) Probability 35.3%, p-value 0.0035

[1] Functional predictions were performed using InterPro and BLAST analysis.
[2] TMD were predicted using TMHMM.
[3] Localisation as assessed from images in the Tryptag database [85], C terminal tag only, na = image not available
[4] In this work, nd = protein not detected in this analysis.
[5] Proteins found in associated with TbMCU in this publication. Y= protein found associated, nd = protein not detected in this analysis.
[6] Proteins listed in the mitochondrial importome defined in this publication. Y= protein listed, nd = protein not detected in this analysis.
[7] Proteins found in IM fraction in this publication. Y= protein found in IM, N= protein not in IM, nd = protein not detected in this analysis.
[8] Protein sequence similarity assessed by PSI-BLAST against sequences in *S. cerevisiae* databases
[9] The top HHpred hit was recorded, unless one of the hits was a known F_1F_o ATP synthase subunit. Also recorded was the hit number (#), the number of amino acids covered by the structural homology and the total number of amino acids of the protein hit in question (x/y), the probability of the hit in % and the p-value of the hit.

REFERENCES

41. Peikert, C. D., Mani, J., Morgenstern, M., Käser, S., Knapp, B., Wenger, C., Harsman, A., Oeljeklaus, S., Schneider, A., and Warscheid, B. (2017) Charting organellar importomes by quantitative mass spectrometry. *Nat. Commun.* 10.1038/ncomms15272

42. Niemann, M., Wiese, S., Mani, J., Chanfon, A., Jackson, C., Meisinger, C., Warscheid, B., and Schneider, A. (2013) Mitochondrial Outer Membrane Proteome of Trypanosoma brucei Reveals Novel Factors Required to Maintain Mitochondrial Morphology. *Mol. Cell. Proteomics*. **12**, 515–528

46. Schädeli, D., Serricchio, M., Ben Hamidane, H., Loffreda, A., Hemphill, A., Beneke, T., Gluenz, E., Graumann, J., and Bütikofer, P. (2019) Cardiolipin depletion-induced changes in the Trypanosoma brucei proteome. *FASEB J.* 10.1096/fj.201901184RR

63. Huang, G., and Docampo, R. (2020) The mitochondrial calcium uniporter interacts with subunit c of the ATP synthase of trypanosomes and humans. *MBio*. 10.1128/mBio.00268-20

84. Sievers, F., Wilm, A., Dineen, D., Gibson, T.J., Karplus, K., Li, W., Lopez, R., McWilliam, H., Remmert, M., Söding, J., Thompson, J.D., and Higgins, D.G. (2011) Fast, scalable generation of high-quality protein multiple sequence alignments using Clustal Omega. *Mol. Syst. Biol.*, **7**, 539

85. Dean, S., Sunter, J. D., and Wheeler, R. J. (2016) TrypTag.org: A Trypansome Genome-wide Protein Localisation Resource. Trends in Parasitology. 10.1016/j.pt.2016.10.009
